# Efficacy of artemether-lumefantrine in treatment of malaria among under-fives and prevalence of drug resistance markers in Igombe-Mwanza, north-western Tanzania

**DOI:** 10.1186/1475-2875-11-58

**Published:** 2012-02-27

**Authors:** Erasmus Kamugisha, Sun Jing, Mercy Minde, Johaness Kataraihya, Gilbert Kongola, Fred Kironde, Göte Swedberg

**Affiliations:** 1 Weill-Bugando University College of Health Sciences, Mwanza, Tanzania; 2 Uppsala University, Uppsala, Sweden; 3 Makerere University, Kampala, Uganda

**Keywords:** *pfcrt*, *pfmdr*1, *pfdhfr*, *pfdhps*, *pfatp*6, Mutations

## Abstract

**Background:**

Drug resistance to anti-malarials is a major public health problem worldwide. This study aimed at establishing the efficacy of artemether-lumefantrine (ACT) in Igombe-Mwanza, north-western Tanzania after a few years of ACT use, and establish the prevalence of mutations in key targets for artemisinin, chloroquine and sulphadoxine/pyrimetamine (SP) drugs.

**Methods:**

A prospective single cohort study was conducted at Igombe health centre using artemether-lumefantrine combination therapy between February 2010 and March 2011. The follow-up period was 28 days and outcome measures were according to WHO guidelines. Blood was collected on Whatman filter paper for DNA analysis. DNA extraction was done using TRIS-EDTA method, and mutations in *Pfcrt*, *Pfmdr*1, *Pfdhfr*, *Pfdhps *and *Pfatp*6 were detected using PCR-RFLP methods established previously.

**Results:**

A total of 103 patients completed the 28 days follow-up. The mean haemoglobin was 8.9 g/dl (range 5.0 to 14.5 g/dl) and mean parasite density was 5,608 parasites/μl. Average parasite clearance time was 34.7 hours and all patients cleared the parasites by day 3. There was no early treatment failure in this study. Late clinical failure was seen in three (2.9%) patients and late parasitological failure (LPF) was seen in two (1.9%). PCR-corrected LPF was 1% and adequate clinical and parasitological response was 96%. The majority of parasites have wild type alleles on *pfcrt *76 and *pfmdr*1 86 positions being 87.8% and 93.7% respectively. Mutant parasites predominated at *pfdhfr *gene at the main three positions 108, 51 and 59 with prevalence of 94.8%, 75.3% and 82.5% respectively. Post-treatment parasites had more wild types of *pfdhps *at position 437 and 540 than pre-treatment parasites. No mutation was seen in *pfatp6 *769 in re-infecting or recrudescing parasites.

**Conclusion:**

The efficacy of artemether-lumefantrine for treatment of uncomplicated malaria is still high in the study area although the rate of re-infection is higher than previously reported. Parasite clearance after 48 hours was lower compared to previous studies. The prevalence of wild type allele *pfcrt *76 K and *pfmdr*1 86 N was high in the study area while markers for SP resistance is still high. Artemether-lumefantrine may be selecting for wild type alleles on both positions (437 and 540) of *pfdhps*.

## Background

Drug resistance to anti-malarials is a major public health problem worldwide [1]. In 2006, Tanzania changed policy from use of sulphadoxine-pyrimethamine (SP) as a first-line drug for treatment of uncomplicated malaria to artemether-lumefantrine combination therapy (AL) [2]. This was a second change following the first change from chloroquine (CQ) to SP in 2001 [3,4]. This milestone is similar in many African countries where malaria is endemic. A change of anti-malarial drug policy has been derived by development of drug resistance to commonly used drugs by the *Plasmodium *parasites, especially *Plasmodium falciparum *which causes more than 90% of infection in sub-Saharan Africa. Although there are reports of decreasing paediatric malaria infection [5,6] and burden of malaria [1]; malaria is still a major public health disease causing 243 million cases every year, of which over 85% are in Africa. Malaria led to 863,000 deaths in 2008 and 89% of them occurred in the sub-Saharan Africa region [1]. Data from the field are now reporting emergence of what is referred to as artemisinin resistance due to increased number of parasites, which shows delayed clearance from blood circulation on artemisinin combination therapy (ACT) [7]. Clinical trials carried out so far in Africa shows high efficacy of AL combination therapy [8-11]. Hunt *et al*, 2009 reports that analysis of studies in East Africa shows that the parasites were being controlled less well by the artemisinin component of ACT in 2007/2008 studies than in 2005/2006 [12]. As these reports are coming up, the mechanism of action of artemisinin is not known yet, a few target genes have been suggested with inconclusive findings [13-16]. Treatment with AL has led to selection of wild type alleles at molecular markers for CQ resistance (*pfcrt *76 K and *pfmdr1 *86 N) with a concomitant reduction in susceptibility to lumefantrine [17]. Even before introduction of AL, re-emergence of wild types for *pfcrt *has been reported in Malawi with restored sensitivity to CQ [18,19]. It is not known whether it is the use of lumefantrine, absence of CQ in the field or both factors acting in synergy that leads to selection of CQ susceptible parasites. Tanzania is a country that has significantly minimized the use of CQ for about 10 years now but continues to use SP for IPTp (intermittent presumptive treatment in pregnancy). In such a situation there is a need to continue monitoring the efficacy of AL in endemic areas and prevalence of molecular markers for drug resistance so as to give evidence-based data to national malaria control programmes. This study aimed at establishing the efficacy of AL in Igombe, Mwanza, north-western Tanzania, after a few years of AL use and establish the prevalence of mutations in key targets for artemisinin, chloroquine and sulphadoxine/pyrimetamine (SP) drugs.

## Methods

### Study area and design

This was an interventional prospective single cohort study conducted at Igombe health centre in the vicinity of Mwanza city in Tanzania. In this area malaria is mesoendemic and the catchment area for Igombe health centre is a semi-urban, with a population of around 40,000 inhabitants.

### Recruitment of patients

Patients with fever, aged between six and 60 months who attended the clinic during the study periods (between February 2010 and July 2010 and between October 2010 and March 2011) were screened for malaria parasites. Detailed medical history, clinical examination and both thick and thin blood films were done after obtaining an informed consent from the parents or guardian. Recruitment was based on inclusion criteria set by WHO [20] (parasitaemia between 2,000 and 200,000 asexual stage parasites/μl, axillary temperature ≥ 37.5°C). Patients must not have history of anti-malarial use in the last 14 days, no signs of severe malaria or danger signs and no other infections.

### Treatment of patients and follow-up

A six-dose regimen of artemether-lumefantrine (Co-artem^®^, Novartis, Basel, Switzerland) was used to treat recruited patients. A first dose was given as a direct observed therapy (DOT) and the next doses was supplied to the parents/caretakers for giving to patients at eight hours from the time of the first dose and morning and evenings of successive two days. For patients weighing from 5 kg to less than 14 kg a single tablet (20 mg/120 mg artemether/lumefantrine) was given, those from 15 kg to less than 24 kg received two tablets, those with 25 to less than 34 kg received three tablets. Patients with fever and axillary body temperature ≥ 38.5°C were given paracetamol and those with anaemia (Hb between 5 and 9 g/dl) were given haematinics. If a patient vomited the treatment drug within 30 minutes the dose was repeated and those who vomited more than once were excluded from the study and changed to parenteral quinine. Patients were followed up on days 1, 2, 3, 7, 14, 21 and 28. Patients were reminded of their visiting dates by mobile phones if the parents owned one, or through the 10 cell-leaders' phones; those who did not turn up on the scheduled days were visited by a member of the research team. A patient was withdrawn from the study if the follow-up was not complete and could not be traced the following day and these included patients who travelled to other places. Also use of other anti-malarial drugs or non-compliance especially to the second dose at eight hours led to withdrawal of patients from the study.

According to WHO *in vivo *test protocol [20] the outcomes were classified into early treatment failure (ETF), late clinical failure (LCF), late parasitological failure (LPF) and adequate clinical and parasitological response (ACPR).

ETF was defined as the occurrence of one of the following signs: signs of severe disease or danger signs on day 1, 2 or 3 with parasitaemia, level of parasitaemia on day 2 that exceeded that on day 0, an axillary temperature of 37.5°C or higher on day 3 in the presence of parasitaemia or a level of parasitaemia on day 3 that was at least 25% of the level at time of enrolment.

LCF was defined as the occurrence (to patients who did not have ETF) of one of the following during days 4 to 28: danger signs, severe malaria or an axillary temperature of 37.5°C or higher in the presence of parasitaemia.

LPF was defined as presence of parasitaemia on any day from day 7 to day 28 without signs of severe disease or fever and not previously meeting criteria of ETF or LCF

ACPR was defined as absence of parasitaemia on day 28 irrespective of axillary temperature without previously meeting any of the criteria of ETF, LTF or LPF

### Ethical clearance

The study was approved by the Joint Weill-Bugando ethical clearance committee and informed consent was obtained from the parents/guardians of all patients.

### Blood sample collection and haemoglobin estimation

On day of recruitment finger prick was done aseptically and blood was spotted on Whatman no 1 filter paper, thick and thin blood smears were done and stained with Giemsa. A venopunture was done and 3 ml of blood was taken in EDTA vacutainer for full blood count (including haemoglobin estimation) using Cell Dyn 3700 machine (Abbot Laboratories USA). On follow-up only, finger prick blood was collected and spotted on filter papers.

### Molecular analysis

DNA was extracted from dried blood spot on filter paper using TRIS-EDTA method [21] and stored at -20°C until future use. Mutations on *pfcrt *position 76 were determined using primers and RFLP performed as explained elsewhere [22]. *Pfmdr1 *gene was also amplified by nested PCR and RFLP done according to protocols published before [23]. For *pfdhfr*, outer PCR was done using primer *pfdhfr1 *and *pfdhfr2 *and nested PCR was done using primer *pfdhfr3 *and *pfdhfr4 *for position 108 and *pfdhfr5 *and *pfdhfr6 *for nested reaction on position 51 and 59 (see Tables 1 and 2 for PCR primers and programmes). The *pfdhps *gene covering positions 540 and 437 was amplified and digested using primers and protocols published previously [24]. For *pfatp6 *positions 263 and 769, PCR-RFLP methods described by Kamugisha *et al*, 2011 and Sisowath *et al*, 2007 were used [25,26]. All PCR products and restriction digests were separated by agarose gel electrophoresis and visualized using UV trans-illuminator. Distinction between re-infection and recrudescence was done using PCR and RFLP on *msp-2 *gene as previously described [27]. Data were analysed using SPSS version 17.

**Table 1:** Primers used for *pfdhfr *amplification

Name	Sequence
Pfdhfr1(outer forward)	5'-ATG ATG GAA CAA GTC TGC GAC-3'

Pfdhfr2(outer reverse)	5'-C TTG ATA AAC AAC GGA ACC TCC-3'

Pfdhfr3(nested forward)	5'-ACT ACA CAT TTA GAG GTC TAG G-3'

Pfdhfr4(nested reverse)	5'-GG TTC TAG ACA ATA TAA CAT TTA TCC-3'

Pfdhfr5(nested forward)	5'-GCC ATA TGT GCA TGT TGT AAG GTT GAA AG-3'

Pfdhfr6(nested reverse)	5'-CAT ATT TTG ATT CAT TCA CAT ATG TTG TAA CTG CTC-3'

**Table 2:** Programme used for *pfdhfr *amplification

Name	PCR programme
Outer	94°C 3 min; 5 cycles:94°C 30 sec, 56°C 30 sec, 72°C 45 sec; 8 cycles: 92°C 30 sec, 55°C 30 sec, 72°C 45 sec; 12 cycles: 92°C 30 sec, 53°C 30 sec, 72°C 45 sec; 18 cycles: 92°C 30 sec, 50°C 30 sec, 72°C 45 sec; 16°C hold

Nested (pfdhfr3+pfdhfr4)	94°C 3 min; 30 cycles:94°C 1 min, 50°C 1 min, 72°C 1 min; 72°C 10 min; 4°C hold

Nested (pfdhfr5+pfdhfr6)	94°C 3 min; 30 cycles: 94°C 1 min, 55°C 1 min, 72°C 1 min; 72°C 10 min; 4°C hold

## Results

**Figure 1:**
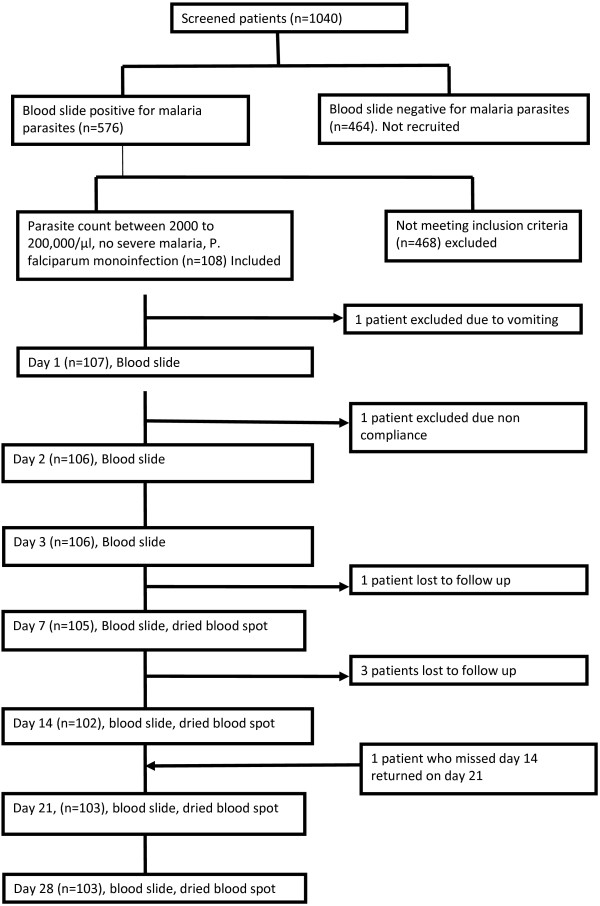
**Patient recruitment and follow-up for 28 days in Igombe**.

A total of 1,040 children under five years of age, with fever, were screened for malaria parasites during the study period. Five hundred and seventy six (55.4%) were found positive for malaria parasites but only 108 met the inclusion criteria and were recruited (Figure 1). Five patients could not complete the study, three of these were lost to follow-up while one was referred due to severe vomiting (vomited the drug twice) and another patient was not compliant as he did not take the dose at eight hours. Therefore 103 patients completed the 28 days follow-up and were analysed.

Males were 52 (51.5%) and females were 51 (49.5%) and the average age was 38.7 months. The mean haemoglobin was 8.9 g/dl (range 5.0 to 14.5 g/dl), and mean parasite density was 5,608 parasites/μl (ranging from 2,000 to 56,800 parasites/μl).

**Figure 2:**
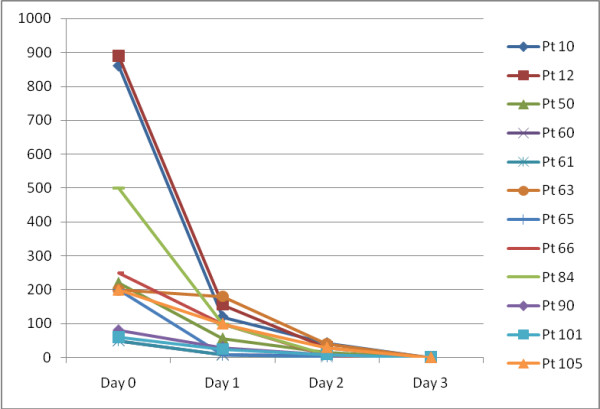
**Parasite clearance in 12 patients who had parasitaemia on day 2**.

Average parasite clearance time was 34.7 hours, 70 (68%) cleared parasites within the first 24 hours and cumulatively 91 (88.4%) patients had cleared the parasites at 48 hours. The remaining 12 (11.7%) patients had positive blood slide at day 2 and they were all cleared of parasites by day 3 (Figure 2). Only one patient had fever on day 2 and others had fever resolution within the first 24 hours.

There was no ETF in this study, LCF was seen in three (2.9%) patients, LPF was seen in two (1.9%) and ACPR was achieved in 98 (95.1%) patients before correction with PCR. PCR corrected LPF was 1% and ACPR was 96% (Table 3).

**Table 3:** PCR corrected and uncorrected cure rate

	No	% Prevalence PCR uncorrected	No	% Prevalence PCR corrected
ETF	0	0.0	0	0.0

LCF	3	2.9	0	0.0

LPF	2	1.9	1	1.0

ACPR	98	95.1	95	96

Total analysis	103		99	

Withdrawal	2		4	

Loss to follow-up	3			

Total	108			

A high number of subpatent infections were detected by PCR (while looking at the drug resistance markers) but not microscopy on both day 14 and day 28 raising the prevalence to 15 (14.6%) and 13 (12.6%) respectively. All subpatent infections were due to re-infections as shown by different *msp2 *patterns.

### Molecular markers for drug resistance

#### Pfcrt 76 position

A total of 90 samples were successfully amplified at position 76 of *pfcrt *gene (success rate 87.4%) and among these, the majority 80 (88.9%) were wild type and only 10 (11.1%) were mutants. On follow-up samples, after treatment with AL, 12 samples were successfully amplified on day 14 and 28, of which a majority, eight (66.7%), were wild type alleles and four (33.3%) were mutant alleles. The difference between pre-treatment samples and re-infections could not be shown to be significant (Table 4).

**Table 4:** Prevalence of mutations in *pfcrt*, *pfmdr1*, *pfdhfr *and *pfdhps *in pre-treatment and post-treatment samples

Position (N)	Mutants (n)	Percentage	*P*-value
***Pfcrt *76**			

Pre-treatment (90)	10	11.1	

Post-treatment (12)	4	33.3	0.22

***Pfmdr1 *86**			

Pre-treatment (95)	6	6.3	

Post-treatment (19)	8	42.1	0.0000

***Pfdhfr*108**			

Pre-treatment (97)	92	94.8	

Post-treatment (29)	21	74.2	0.0009

***Pfdhfr *51**			

Pre-treatment (97)	73	75.3	

Post-treatment (29)	20	69.0	0.025

***Pfdhfr *59**			

Pre-treatment (97)	80	82.5	

Post-treatment (29)	15	51.7	0.0000

***Pfdhps *437 (N)**			

Pre-treatment (96)	71	74	

Post-treatment (21)	7	33.3	0.0003

***Pfdhps *540**			

Pre-treatment (95)	66	69.5	

Post-treatment (21)	8	38.1	0.0000

#### Pfmdr1 86 position

At this position the wild type were higher, 89 (93.7%) (including 85 pure wild types and four mixed, with wild types dominating) and pure mutants were only six (6.3%). Among the 19 successfully amplified post-treatment samples, the wild type alleles were still in majority at 11 (57.9%) but the proportion of mutants had increased to 42.1%, which is a significant change (Table 4). All mutants in post-treatment samples did not come from same patients with mutation at day 0.

#### Pfdhfr position 108, 51 and 59

For *pfdhfr*, successful amplification was achieved in 97 samples (success rate 97.2%). At position 108, 92 (94.8%) were mutant while four (4.3%) were wild type and one (1.1%) was a mixed infection.

At position 51, the majority 73 (75.3%) were mutant while 12 (12.4%) were wild type and the other 12 (12.4%) were mixed with more mutants.

At position 59, 80 (82.5%) were mutant and nine (9.3%) were wild type, while eight (8.2%) were mixed with more mutants. The prevalence of triple mutants in *pfdhfr *gene in pre-treatment samples was high with 59 samples (64.1%) while 27 (29.4%) had double mutation with various combinations as illustrated in Table 5.

**Table 5:** Prevalence of *Pfdhfr *mutations at position 108, 51, 59

N = 92 Pre-treatment	n	%
Triple mutants	59	64.1

Double mutants 108/51	10	10.9

Double mutants 108/59	2	2.2

Double mutants 59/51	1	1.1

Double mutants 108 with 59/51 and mix at third position	15	16.3

Single mutants	5	5.4

**N = 23 Post-treatment**		

Triple mutants	13	56.5

Double mutants 108/51	2	8.7

Double mutants 108/59	2	8.7

Double mutants 59/51	0	0

Double mutants 108 with 59/51 and mix at third position	3	13.0

Single mutants	3	13.0

In post-treatment samples, mutants were predominant but at a lower percentage than at day 0: 21/29 (74.2%), 20/29 (69%) and 15/29 (51.7%) on position 108, 51 and 59 respectively. In the post-treatment samples the triple mutants were 13/29 (44.8%) and double mutants were 7/29 (24.1%) (Table 5). About eleven patients (47.8%) were mutants in both pre-and post-treatment samples.

#### Pfdhps gene position 540 and 437

A total of 95 samples were successfully amplified at position 540 and 66 (69.5%) were mutants, while 15 (15.8%) were wild type and 14 (14.7%) were mixed with more mutants. At *pfdhps *437, 96 samples were successfully amplified and 71 (74.0%) were mutants while 15 (15.6%) were wild type and 10 (10.4%) were mixed (with mutants predominating). Double mutants in *pfdhps *were 65 (68.4%) out of 95 samples, which amplified at both positions. With the exception of one sample, all mutants at position 540 were also mutants at position 437. There were more wild types at both positions in *pfdhps *in the follow-up samples at day 14 and 28 after treatment with ACT. Prevalence of wild types were 10/17 (58.8%) and 13/21 (61.9%) at positions 437 and 540 respectively.

#### Pfatp6 position 263 and 769

There was no mutation in *pfatp6 *at position 263 in either pre-treatment or post-treatment samples. At position 769, mutations were screened only in re-infection and recrudescing parasites and no mutation was detected in these follow-up samples.

## Discussion

The efficacy of AL in this study area was high at 95.1% (PCR uncorrected) and 96% PCR corrected. This is similar to what has been found in other places of Tanzania and Sub-Saharan Africa in general where cure rates ranged from 96 to 100 in children [8-11,28]. The percentage of re-infection in this study was higher compared to the findings in the above studies. However, a more recent study, Ngasala *et al*, showed similar high frequencies of LPF as shown here [29]. The meeting proceedings reported by Hunt *et al *[12], presented evidence of parasites in East Africa being less well controlled in 2007/2008 studies compared to studies in 2005/2006. This may be the beginning of what is referred to as reduced efficacy of artemisinin combination therapies seen in South-east Asia [7]. In this study recrudescence was low 1%. The mean parasite clearance time in this study was 34.7 hours, which is consistent with what was reported in 2005[8]. The percentage of patients that had cleared the parasites by 48 hours was lower compared to previous studies; this may be an indicator of emergence of the resistant, dormant parasites reported in South-east Asia. The fact that no parasites were extended beyond day 3 shows that this ACT is still more efficacious in Africa than has been seen in South-east Asia. This may be explained by proper use of ACT and relatively short time of use compared to South-east Asia. The main combination in South-east Asia is artemisinin-mefloquine, which may explain some of the difference.

The prevalence of parasites with wild type alleles (*pfcrt *76 K and *pfmdr*186N) is high in this study area compared to other studies done previously in Tanzania. This is a good indicator of return of CQ-sensitive parasites as was shown in Malawi study where Laufer *et al*, found good *in vivo *sensitivity to CQ following a study that revealed decline in mutants (*pfcrt*76T) at this position [19]. The findings in this study could be due to both proper control of CQ [30] or the selection of wild type by use of AL therapy [17]. This adds to the evidence that reduction in the drug pressure in malaria parasites leads to re-emergence of wild type, which has been documented [18]. The decline seen in Tanzania seems to be similar to what was seen in Malawi but differs from what is seen in Kenya and Uganda, the immediately neighbouring countries in the northern part of Tanzania; this is attributed to continued use of certain amounts of CQ in these countries [30]. In Gabon, the prevalence of *pfcrt *mutations was reported to be high more than five years after discontinued use of CQ [31]. In this case, the high prevalence could be explained by the inclusion of amodiaquine in artemisinin-based treatment. There was however, a surprisingly high frequency of mutations in *pfcrt *and *pfmdr1 *in subpatent re-infections found in follow-up samples. This contrasts with most earlier studies, but a similar tendency for *pfcrt *is observed in Ngasala *et al*, where at least no significant difference in *pfcrt *frequencies were found in re-infections [29]. The remaining *pfcrt *and *pfmdr1 *mutations indicates that it will be very difficult to completely reverse CQ resistance once the resistance has been established and reusing CQ may lead to rapid emergence of CQ resistance. Any attempt to reuse CQ should only be done under proper controls where the in vitro susceptibility can be monitored.

On the other hand the prevalence of triple and double *pfdhfr *mutants in Mwanza is very high 64.1% and 29.4% compared to that reported previously in this area [3]. This could be explained by the continued use of SP in IPTp and probably in the treatment of malaria as the change of policy from SP to ACT did not go hand in hand with the ban of SP as occurred with CQ. The prevalence of triple and double mutants in this place creates the doubt on the usefulness of continued use of these drugs in IPTp but also removes hope of the possibility of combining these drugs with artemisinins. However, in re-infections lower level of mutants were seen for *pfdhfr *and more dramatically for *pfdhps *indicating a possible rapid return to SP susceptibility with continued use of AL.

## Conclusions

The efficacy of artemether-lumefantrine for treatment of uncomplicated malaria was still high in the study area although the rate of re-infection was high. There was a high number of patients who got subpatent malaria infection after treatment with ACT, which was diagnosed by PCR later at day 14 or 28. The prevalence of *pfcrt *76 K and *pfmdr1 *86 N was high in the study area while markers for SP resistance shows that the resistance to SP may still be very high and even increasing. Artemether-lumefantrine may be selecting for wild type alleles on both positions (437 and 540) of *pfdhps*

## Competing interests

The authors declare that they have no competing interests.

## Authors' contributions

EK, SJ and GS carried out the molecular genetic studies and drafted the manuscript. MM, JK and EK carried out field study, patients follow-up and drafted the manuscript. GK, FK and GS conceived the study, and participated in its design and coordination. All authors read and approved the final manuscript.
